# Correlation Between Bacterial Diversity and Flavor Compounds in Hengshui Laobaigan Baijiu

**DOI:** 10.3390/foods15111922

**Published:** 2026-05-29

**Authors:** Xuelian Yang, Ying Hu, Rui Zhang, Jingchao Li

**Affiliations:** Beijing Engineering and Technology Research Center of Food Additives, Beijing Technology and Business University (BTBU), Beijing 100048, China; huying18325605102@163.com (Y.H.); 17274865680@163.com (R.Z.); 14790629585@163.com (J.L.)

**Keywords:** high-throughput sequencing, laobaigan baijiu, bacterial diversity, flavor compounds, correlation

## Abstract

The fermentation cycle critically influences flavor formation in Laobaigan Baijiu, but its effect on the bacterial community–flavor compound correlation remains unclear. This study investigated the co-variation between bacterial community turnover and flavor changes across distinct fermentation rounds of Hengshui Laobaigan Baijiu. Bacterial community structure was characterized via high-throughput sequencing (HTS), while volatile flavor compounds were profiled using headspace solid-phase microextraction combined with gas chromatography–mass spectrometry (HS-SPME–GC–MS). Partial least squares regression (PLSR) revealed their correlation. The analysis revealed 45 volatile compounds in total, with the **D-2–D-3** stages serving as the peak production period and stages **E-5–E-6** as the key formation period for esters. *Lactobacillus* dominated the early Dacha (first batch) fermentation, whereas *Pseudomonas*, *Chryseobacterium*, and *Delftia* became dominant after Jiuqu addition in Ercha (second batch). *Sphingomonas* and *Pseudomonas* were the major flavor contributors, showing very strong correlations with 9 and 6 compounds, respectively. *Sphingomonas* was positively correlated with nine compounds (e.g., isoamyl acetate, ethyl pentanoate, acids, benzaldehyde), while *Pseudomonas* was positively correlated with six esters (e.g., ethyl acetate, ethyl butyrate). *Delftia*, *Pantoea*, and *Lactobacillus* also played important roles in volatile formation. This study establishes a correlation network between bacterial communities and flavor compounds in a multi-round solid-state fermentation system, offering a methodological reference for flavor regulation not only in Laobaigan Baijiu but potentially in other traditional fermented foods with similar batch fermentation processes. This study provides theoretical support for the optimization of Laobaigan Baijiu fermentation and the enhancement of final Baijiu quality.

## 1. Introduction

Chinese Baijiu is known as the national liquor of China [1]. Baijiu can be easily differentiated from other liquors due to its distinctive flavor, and it can be classified into 12 different types based on its distinctive flavor characteristics, that is, strong, soy sauce, light, rice, Feng, Te, sesame, Laobaigan, Fuyu, herbal, Chi, and Jian [2]. Laobaigan-flavor Baijiu is mainly produced in northern and northeastern China, represented by the Laobaigan Baijiu from Hengshui City, Hebei Province, which is renowned for its unique aroma, distinctive fermentation process, and characteristic flavor profile [3]. Notably, the Laowuzeng multi-round batch fermentation process used for Laobaigan Baijiu shares core characteristics with the production of other well-known fermented foods, such as the continuous batch fermentation in other Baijiu flavor types [4,5] and the stacked fermentation of traditional vinegar [6]. Therefore, Laobaigan Baijiu serves as an excellent model to study how fermentation rounds shape bacterial succession and flavor development, with findings that may be transferable to other solid-state fermentation systems. Hengshui Laobaigan Baijiu is produced from sorghum using medium-temperature Daqu (traditional starter culture) as the saccharifying and fermenting agent. It employs the Laowuzeng process, a continuous batch distillation method with mixing of fresh and fermented grains. The production involves fermentation in underground earthen jars, simultaneous steaming and mixing of materials, fractional collection of distillate, graded storage, and meticulous blending. This process is characterized by a short fermentation cycle, high liquor yield, and a brief storage period [7]. Laowuzeng (or repeated-batch fermentation) is a distinctive Baijiu-making technique. In this process, the fermented grains are not directly distilled; instead, fresh grains are added to the fermented grains prior to distillation [8]. Therefore, this method influences the quantity and diversity of flavor compounds in the final product. Dacha fermented grain is generated by mixing distiller’s yeast with starting materials for the initial fermentation, after which Dacha Baijiu is acquired via distillation of these fermented grains. For maximizing the utilization of starting materials, fresh distiller’s yeast is introduced into the Dacha residue following distillation. That solid matrix, which proceeds through a subsequent fermentation, turns into Ercha fermented grain, and Ercha Baijiu is then acquired via distillation [9].

The widespread application of high-throughput sequencing (HTS) together with gas chromatography–mass spectrometry (GC–MS) has been driven by the rapid development of modern molecular biology and analytical technologies, enabling studies into microbial diversity and flavor compounds over the course of Baijiu fermentation, thereby improving our understanding of microbial community composition and characteristic flavor profiles [10]. The microorganisms in Daqu are not only involved in the major metabolic pathways of Baijiu production but also generate certain flavor compounds or their precursors, thereby enhancing Baijiu’s flavor characteristics while playing a major role in generating its flavor constituents [11]. Previous studies have revealed the role of fungi and their association with flavor formation during Laobaigan Baijiu fermentation. However, the correlation linking bacterial communities to flavor compounds in Laobaigan Baijiu has not been systematically examined, as bacterial communities are often more complex than fungal communities [12]. Yang et al. analyzed the correlation between bacterial community succession and changes in flavor compounds during whey wine fermentation and found that *Lactobacillus* and *Lactococcus* were the dominant bacteria as fermentation progressed [13] Xue et al. [14] evaluated bacterial diversity plus flavor traits in Dacha relative to Ercha fermented grains within Baijiu of the Fen flavor type and found that Ercha had significantly higher bacterial diversity than Dacha. In Dacha, *Lactobacillus* served as the dominant genus, whereas in Ercha, *Pseudomonas* and *Bacillus* were the dominant genera. Studies have also shown that bacteria may be the driving force of microbial succession during Daqu production [15].

This study employed HTS to analyze bacterial diversity and community structure dynamics at different fermentation stages of Laobaigan Baijiu, used HS-SPME–GC–MS to detect volatile flavor compounds during fermentation, and applied PLSR to explore the correlation between changes in bacterial community structure and the formation of flavor compounds. Although this study focuses on Hengshui Laobaigan Baijiu, the analytical framework and the observed co-variation patterns—such as the shift from Lactobacillus dominance in the first batch to Pseudomonas activation in the second batch—may provide insights into microbial flavor regulation in other solid-state fermented foods, especially those employing multi-round batch fermentation processes [4,5,6,16,17]. This work thus offers a theoretical foundation for controlling processing and enhancing the quality of characteristic flavor compounds in Laobaigan Baijiu.

## 2. Materials and Methods

### 2.1. Sample Collection

Sampling was performed at a Laobaigan Baijiu distillery located in Hengshui (Hebei Province). Batches produced using the typical Laowuzeng continuous batch fermentation process in underground earthen jars were selected. Six fermentation time points (0, 15, 30, 45, 60, and 75 days) were chosen, and the samples were sequentially designated as **D-1** through **D-3** and **E-4** through **E-6**, with the former three (**D-1** to **D-3**) representing Dacha fermentation and the latter three (**E-4** to **E-6**) representing Ercha fermentation. For each time point, five independent biological replicates were collected from five separate earthen jars. Within each jar, five sampling points were taken from the upper, middle, and lower layers of the fermented grains (15 points in total) and mixed to obtain one composite sample. Thus, five composite samples were obtained per time point. All samples were stored at 4 °C for subsequent HTS and flavor compound analysis.

### 2.2. Extraction of DNA, PCR Amplification, and Sequencing

Genomic DNA was extracted using the E.Z.N.A.™ Mag-Bind Soil DNA Kit (Merck, Darmstadt, Germany). The DNA concentration was diluted to 10–20 ng/μL to meet sequencing requirements. The V3–V4 hypervariable region of the bacterial 16S rRNA gene was amplified using primers 341F (5′-CCTAYGGGRBGCASCAG-3′) and 806R (5′-GGACTACNNGGGTATCTAAT-3′). The primers were synthesized by Sangon Biotech (Shanghai, China).The PCR program was as follows: initial denaturation at 94 °C for 5 min; 30 cycles of denaturation at 93 °C for 30 s, annealing at 55 °C for 30 s, and extension at 72 °C for 1 min 30 s; followed by a final extension at 72 °C for 10 min. The PCR reaction mixture (17.2 μL) contained 1 μL of template DNA, 2.5 μL of 10× buffer, 2 μL of dNTPs, 0.3 μL of Taq polymerase, 1 μL of each primer, and ddH_2_O to a final volume of 17.2 μL. All PCR reagents (10× buffer, dNTPs, and Taq polymerase) were purchased from Takara Bio (Kusatsu, Shiga, Japan).The purified PCR products were used for library construction and paired-end sequencing on an Illumina HiSeq PE250 platform (2 × 250 bp, Illumina, Inc., San Diego, CA, USA). Raw data were saved in FASTQ format. QIIME software (version 1.9.1) was employed to trim primer sequences, remove non-specific sequences, and cluster sequences at 97% similarity to obtain high-quality operational taxonomic units (OTUs) for subsequent bacterial community diversity analysis.

### 2.3. Analysis of Volatile Flavor Compounds

First, 5 g of fermented grain samples were combined with 20 mL of sterile saline, mixed, and allowed to soak overnight. After ultrasonication (0 °C, 30 min), centrifugation was performed (4 °C, 8000 rpm, 10 min). The clear supernatant was filtered across a 0.22 μm organic membrane and then stored at −20 °C until use. For analysis, 7980 μL of the treated sample was placed into a headspace vial together with 20 μL of 4-methyl-2-pentanol (internal standard) and 5 g of NaCl. Five biological replicates were prepared for each fermentation time point. The SPME fiber used was a 50/30 μm divinylbenzene/carboxen/polydimethylsiloxane (DVB/CAR/PDMS) fiber (Supelco, Bellefonte, PA, USA). After 50 min of headspace extraction, the SPME fiber was introduced into the GC–MS injection port and subjected to desorption lasting 5 min. Subsequently, the GC–MS system was started to collect data for volatile compound analysis.

The GC–MS system was equipped with a DB-WAX capillary column (30.0 m × 0.25 mm × 0.25 μm, Agilent, Santa Clara, CA, USA). Splitless injection mode was applied, and the injection volume was set to 1 μL. The column temperature program was as follows: hold at 35 °C for 2 min, ramp at 2 °C/min to 100 °C and hold for 5 min, then ramp at 10 °C/min to 230 °C and hold for 2 min, and finally ramp at 10 °C/min to 280 °C and hold for 3 min. The mass spectrometer was operated at an ionization voltage of 70 eV using full-scan acquisition across the m/z range from 30 to 350. Quadrupole temperature was set to 150 °C. Volatile compounds were identified by comparing mass spectra with the NIST library and published references. Quantification was performed using the internal standard method with 4-methyl-2-pentanol (final concentration 51.15 μg/L). The concentration of each compound was calculated as: concentration (μg/L) = (peak area of compound × mass of internal standard (μg))/(peak area of internal standard × sample volume (L)). Because full calibration curves were not established for each compound, the reported concentrations are semi-quantitative estimates.

### 2.4. Data Analysis

QIIME software (version 1.9.1) was used for sequence quality control, feature table construction, and taxonomic annotation of the bacterial 16S rRNA gene sequencing data, yielding the bacterial community composition and a relative abundance matrix. Based on the sequencing data, α-diversity indices (Chao1, Shannon, Simpson, etc.) were calculated. To visualize the overall differences and clustering patterns of bacterial community structures across different fermentation stages, principal coordinate analysis (PCoA) with weighted UniFrac distances was carried out. Hierarchical clustering analysis was also conducted based on the relative abundance of bacteria at the genus level. TBtools-II was employed to generate hierarchical clustering heatmaps of volatile flavor compound contents at different fermentation stages. Finally, PLSR (partial least squares regression) was conducted via Simca-14.1 software (SartoriusStedim, Gottingen, Sweden), with proportional abundances of predominant bacterial genera (at genus rank) serving as independent variables and volatile flavor compounds as dependent variables. This analysis constructed a model linking the bacterial community to flavor compounds, elucidated their correlations, and screened for key microorganisms influencing flavor formation in Laobaigan Baijiu.

## 3. Results and Discussion

### 3.1. Bacterial Community Diversity of Hengshui Laobaigan Baijiu

#### 3.1.1. Alpha Diversity Analysis of Bacterial Community

Using bacterial 16S rRNA gene sequencing, a total of 632,920 high-quality sequences were obtained from 30 samples, with sequence lengths ranging from 200 bp to 500 bp. Comprehensive analysis of bacterial sequences from fermented grain samples collected at six different fermentation stages led to the identification of 360 operational taxonomic units (OTUs). As shown in Table 1, observed species and Chao1 reflect species richness, while Shannon and Simpson indices integrate community richness as well as evenness. The sequencing coverage of all samples exceeded 0.90, indicating that the effective sequencing depth was high and that the data accurately reflected the actual structure of the entire microbial consortium [18]. With respect to community species diversity, Laobaigan’s observed species, Chao1, Shannon, and Simpson indices exhibited a downward tendency across both Dacha and Ercha stages. However, the overall bacterial community richness and diversity were higher in the Ercha stage than in the Dacha stage, with the highest values at **E-4** and the lowest at **D-3**. At the early stage of Dacha fermentation (**D-1**), all indices were high, reflecting a nutrient-rich and mild environment that facilitated the rapid activation of various Daqui-derived bacteria and resulted in a complex community structure [19]. As fermentation advanced to the final Dacha stage (**D-3**), the selective pressures of high acidity, high ethanol, and extreme anaerobic conditions allowed only a few tolerant dominant bacteria to survive, leading to the lowest diversity of the entire Dacha process [20]. At the early Ercha stage (**E-4**), the diversity indices were comparable to, or even slightly higher than, those at the early Dacha stage, indicating an extremely rich community. Diversity declined at the mid-phase (**E-5**) yet stayed comparatively high. During the last Ercha phase (**E-6**), diversity hit its lowest point across the Ercha process, while still being markedly higher than that at the final Dacha phase (**D-3**). Overall, the richness and diversity indices at each fermentation stage were significantly higher in Ercha than in the corresponding stages of Dacha (*p* < 0.001), and Ercha maintained a relatively high level of diversity throughout, which may be attributed to differences in raw material composition, initial microbiota, and environmental buffering capacity between the two fermentation rounds.

**Figure 1:**
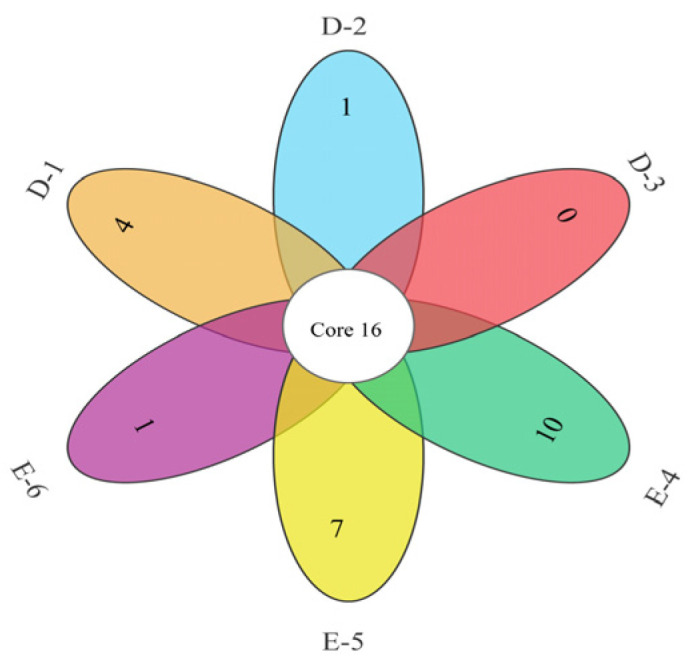
Petal plot of bacterial OTU distribution across fermentation stages.

Meanwhile, as shown in Figure 1, 16 shared OTUs were identified across different fermentation stages of Laobaigan Baijiu, representing a core microbiota that persisted throughout both Dacha and Ercha. In the early fermentation stage, **D-1** exhibited the highest number of unique OTUs, indicating that this stage was the most differentiated phase of the microbial community in Dacha fermentation, possibly corresponding to key functional microbiota involved in raw material saccharification and initial fermentation. By stage **D-2**, the OTU count declined rapidly. The Ercha group possessed substantially more unique OTUs than the Dacha group, reflecting a marked increase in microbial diversity and specificity during the Ercha stage. At **E-4**, the number of unique OTUs quickly increased to 10, making this the stage with the richest functional microbiota in Ercha fermentation. Subsequent alignment of each OTU against a bacterial taxonomic database enabled species annotation, resulting in the identification of 13 phyla and 20 genera of bacteria.

#### 3.1.2. Sample Similarity Analysis

**Figure 2:**
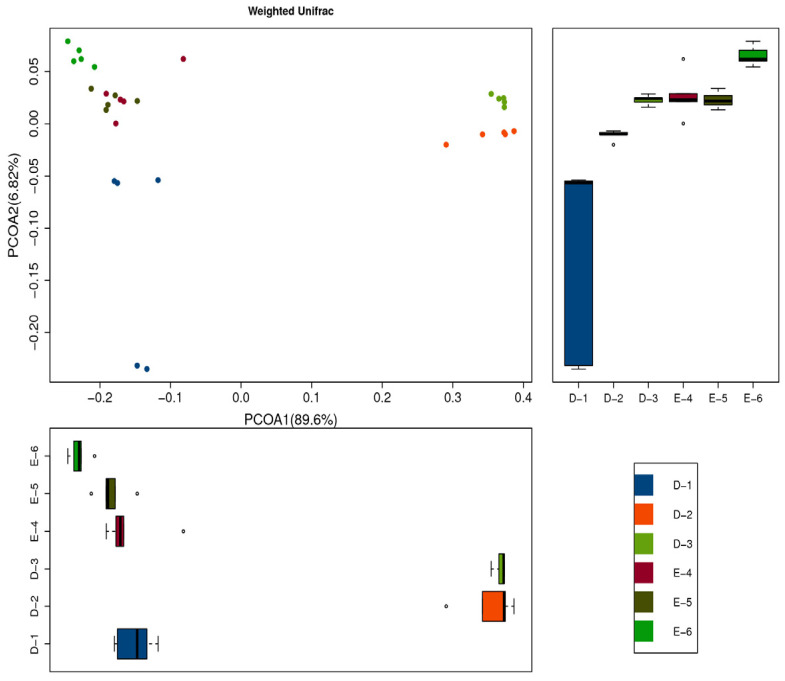
PCoA of bacterial communities across different fermentation stages of Laobaigan Baijiu.

**Figure 3:**
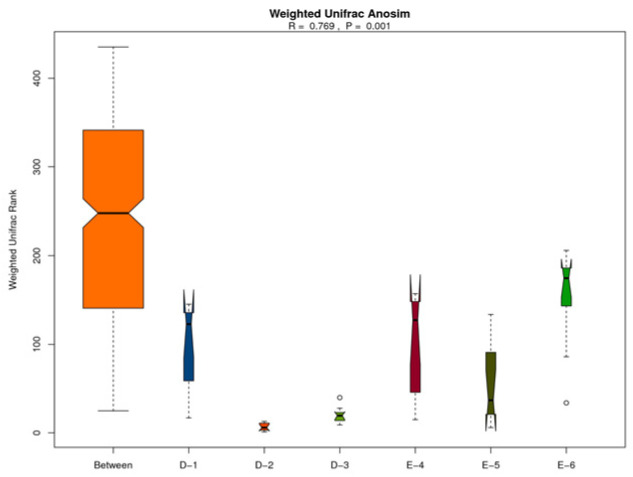
ANOSIM analysis of bacterial communities based on weighted UniFrac distances among the six fermentation stages.

Using weighted UniFrac distances, principal coordinate analysis (PCoA) assessed β-diversity across samples. The PCoA results showed that the first and second principal components explained 89.6% and 6.82% of the total variance, respectively (Figure 2). Along the first principal coordinate, which explained 89.6% of the variance, **D-2** and **D-3** clustered together, while **D-1** together with **E-4**, **E-5**, and **E-6** formed another cluster, with **E-4** and **E-5** being particularly close. Along the second principal coordinate (explaining 6.82% of the variance), **D-3**, **E-4**, and **E-5** were close to each other, whereas **D-1** exhibited a wide spread, indicating large within-group variation. Integrating the first and second principal coordinates, **D-2** and **D-3** exhibited similar sample compositions, as did **E-4** and **E-5**. Notably, **D-1** showed large within-group variation, indicating that the composition of fermented grain samples was unstable at the early fermentation stage. All Dacha samples were spatially separated from all Ercha samples in the PCoA plot, with no overlap between the two groups. PERMANOVA (Adonis, 999 permutations) based on the same weighted UniFrac distance matrix confirmed significant differences among the six fermentation stages (R^2^ = 0.646, *p* = 0.001). Furthermore, ANOSIM based on weighted UniFrac distances (999 permutations) further confirmed a significant difference among the six fermentation stages (R = 0.795, *p* = 0.001; Figure 3), indicating that between-group dissimilarity exceeded within-group dissimilarity. His clustering pattern indicates significant differences in bacterial species and their abundances between Dacha and Ercha [21].

**Figure 4:**
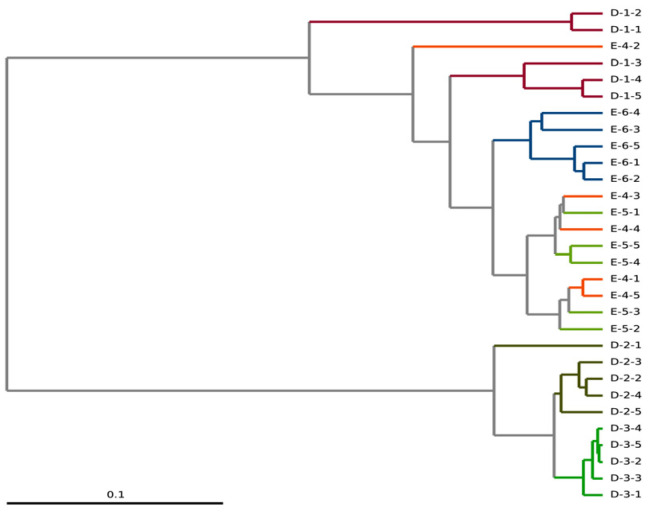
Hierarchical clustering of genus-level bacterial communities based on weighted UniFrac distance.

Hierarchical cluster analysis at the genus level further revealed similarities in bacterial community structure among samples from different fermentation stages (Figure 4). The results showed that samples at **D-1** clustered together with some samples at **E-4**, suggesting that the bacterial community at the early Dacha stage (saccharification stage) shared some similarity with that at the early Ercha stage (**E-4**). Samples at **D-2** formed a continuous independent cluster, representing the core functional microbiota at the mature stage of Dacha fermentation. Samples at **D-3** formed a separate cluster and were separated from all Ercha samples (**E-4**, **E-5**, **E-6**) by the intact **D-2** branch, with no branch crossing. This indicates that the bacterial community structure at the end of Dacha fermentation had undergone substantial succession and was clearly distinct from that of Ercha fermentation samples. During the Ercha fermentation period, samples at **E-6** formed a continuous cluster, whereas those at **E-4** and **E-5** were intermingled and tightly clustered, with very short intra-group distances. This indicates an extremely high similarity in bacterial community structure across different time points of Ercha fermentation, which may constitute the microecological basis for the more uniform flavor characteristics and more efficient flavor compound generation in Ercha Baijiu.

### 3.2. Bacterial Community Composition of Hengshui Laobaigan Baijiu

**Figure 5:**
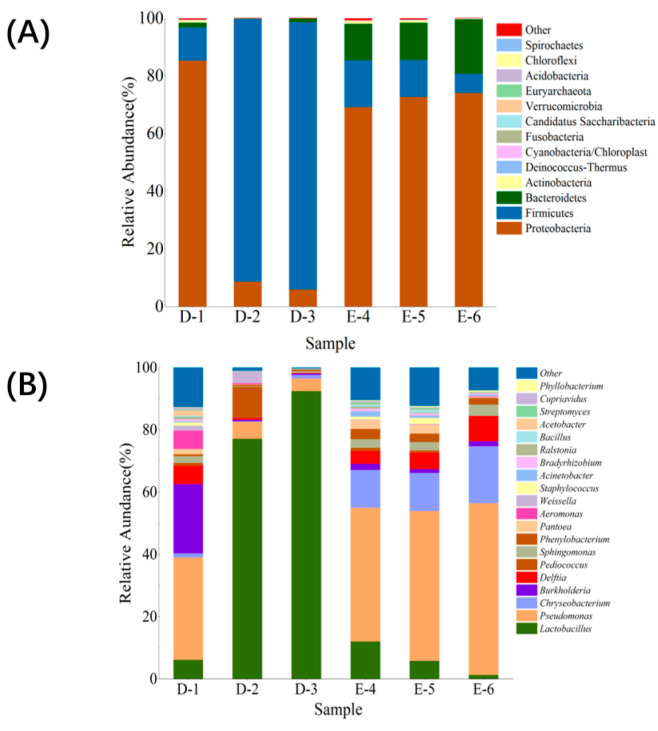
Bacterial community relative abundance across various fermentation phases of Laobaigan Baijiu. (**A**) Phylum level; (**B**) genus level.

To explore the reasons for the changes in bacterial diversity during the fermentation of Hengshui Laobaigan Baijiu, a taxonomic analysis was performed to compare bacterial community structures at the phylum and genus levels across different fermentation time points. As shown in Figure 4, the bacterial species identified in the fermented grain samples from the six fermentation stages belonged to 13 phyla and were further assigned to 20 genera. A small proportion of unclassified microorganisms with low relative abundance were collectively grouped under “others”. Regarding phylum-level classification, the predominant bacterial phyla included *Proteobacteria*, *Firmicutes*, and *Bacteroidetes*, together with Actinobacteria (Figure 5A). Among these, Proteobacteria and Firmicutes were overwhelmingly dominant throughout the fermentation process, yet they exhibited opposite trends. Proteobacteria showed very high relative abundances in samples **D-1** together with **E-4**, **E-5**, and **E-6**, reaching an absolute dominance of 85.38% in **D-1**. In contrast, the proportion of Firmicutes increased markedly in **D-2** and **D-3**, accounting for 91.05% and 92.75%, respectively. Most bacteria within the phylum Firmicutes, including *Lactobacillus* and *Lactococcus*, are anaerobes capable of adapting to highly acidic and oxygen-limited environments [22]. Previous studies on the bacterial community and succession dynamics of Daqu for sesame-flavor Baijiu have also revealed that Firmicutes was the dominant bacterial phylum [23]. Firmicutes are generally closely linked to carbohydrate metabolism and the production of short-chain fatty acids (e.g., lactic acid and butyric acid), whereas certain genera within Proteobacteria (e.g., *Acetobacter*) participate in acetic acid metabolism. The inverse changes in the abundances of these two phyla may reflect differences in fermentation intensity or oxidative conditions among the samples [24]. In the Ercha stage, samples **E-4**, **E-5**, and **E-6** were all overwhelmingly dominated by Proteobacteria, with a stable proportion of Bacteroidetes, and the bacterial community structure became more uniform. Previous studies on other Baijiu flavor types have also revealed that Firmicutes and Proteobacteria dominate the bacterial community during Daqu fermentation [25,26].

According to genus-level analysis, four dominant genera were found among the samples: *Lactobacillus*, *Pseudomonas*, *Chryseobacterium*, and *Delftia* (Figure 5B). The bacterial community structure clearly differed between the Dacha fermentation (**D-1**, **D-2**, **D-3**) and the Ercha fermentation (**E-4**, **E-5**, **E-6**), with significant differences in the composition and relative abundances of the dominant genera. *Lactobacillus* and *Pseudomonas* were the core dominant genera in Dacha and Ercha, respectively. The **D-1** sample exhibited a complex community composition, with multiple genera—including *Lactobacillus*, *Pseudomonas*, and *Burkholderia*—coexisting. At this stage, flavor precursors had accumulated abundantly, whereas characteristic flavors had not yet developed. During the **D-2** and **D-3** stages of fermentation, *Lactobacillus* abundance rose sharply, attaining 92.48% at **D-3** and thereby emerging as the overwhelmingly dominant genus. In contrast, the relative abundances of *Pseudomonas*, *Chryseobacterium*, *Burkholderia*, and *Delftia* decreased. However, during the Ercha fermentation stage, the abundance of *Lactobacillus* was substantially lower than that in Dacha, and it no longer dominated the community. Studies have shown that almost all bacteria in Dacha belong to the genus *Lactobacillus* (phylum Firmicutes). These bacteria can produce various antibacterial substances and thus inhibit other bacteria [27]. In samples from fermentation stages **E-4** to **E-6**, *Pseudomonas* was the overwhelmingly dominant genus, with relative abundances ranging from 42.95% to 55.15%. *Chryseobacterium* was the subdominant genus during the Ercha stage. *Burkholderia* and *Delftia* were also consistently detected throughout the Ercha fermentation, representing important components of the Ercha bacterial community, although their abundances differed from those observed in Dacha. The production of large amounts of lactic acid in Daqu creates an acidic environment that inhibits the growth of acid-sensitive bacteria, whose presence may be detrimental. Therefore, the bacterial community succession during this period facilitates the selection of beneficial bacteria [28].

### 3.3. Flavor Compound Changes in Hengshui Laobaigan Baijiu

A total of 45 flavor compounds were detected throughout the fermentation process (see Table 2), comprising 10 alcohols, 17 esters, 6 aldehydes, 4 ketones, and 3 acids, with the remaining five categories (furans, acetals, aromatic compounds, lactones, and sulfur-containing compounds) each present as a single compound. These aroma compounds accumulated during fermentation and reached their highest levels at the end of the process.

The volatile flavor compounds produced during fermentation can also reflect changes in microbial species and their abundances at different fermentation stages [29]. Headspace solid-phase microextraction coupled with GC-MS (HS-SPME-GC-MS) served to determine volatile flavor compound profiles during Hengshui Laobaigan Baijiu fermentation. Table 2 reveals that alcohols and esters dominated, contributing 67.55% and 9.73% to total flavor compounds, respectively, while each remaining flavor category accounted for less than 7% of the overall content. At the **D-1** stage, immediately after Daqu addition, the microorganisms were in the lag phase of their growth cycle. During adaptation to the environment, essentially no flavor compounds were produced. Subsequently, as fermentation progressed to the **D-2** stage, compounds such as ethyl acetate, acetals, 2-methylpropanal, and 2,3-butanedione began to be synthesized in large quantities, along with small amounts of alcohols. At the **D-3** stage, alcohols and esters continued to increase substantially, whereas aldehydes and ketones began to decrease. As fermentation progressed into the Ercha stage, the addition of fresh grains (Ercha input) together with Daqu led to further accumulation of alcohols. By the **E-4** stage, ethyl acetate, ethyl hexanoate, and other esters showed a slight decrease due to biochemical reactions such as microbial transformation or esterification. However, at the end of fermentation (**E-6**), ethyl acetate and ethyl hexanoate reached their highest levels among all stages. Esters critically shape the flavor of Baijiu. For Laobaigan-flavor Baijiu, ethyl lactate and ethyl acetate serve as the primary ester species; their concentrations and mutual ratio strongly influence the style features of this Baijiu [30,31]. Ethyl acetate is an important fruity aroma compound. A typical Laobaigan-flavor style is attainable solely when ethyl acetate, together with ethyl lactate, each fall within a suitable concentration range and when the ratio (ethyl lactate/ethyl acetate) meets or exceeds 0.8 [1]. Previous studies have reported that the volatile compounds in light-flavor Daqu are predominantly esters and alcohols, including ethyl butyrate, 1-hexanol, 1-octen-3-ol, and phenylethyl alcohol, all of which occur at relatively high abundances. Laobaigan-flavor and light-flavor Baijiu are both northern Chinese Baijiu, and their Daqu production processes (using medium-temperature Daqu) are similar [32,33]. Aldehydes and ketones play a significant role in Baijiu flavor. During fermentation, glycolysis takes place, and the microbial degradation of starch compounds in raw materials generates phenylethyl alcohol (2-phenylethanol). Both phenylethyl alcohol and phenylacetaldehyde possess a rose-like aroma [34,35]. Acetaldehyde is an intermediate product of microbial metabolism during fermented grain fermentation. The condensation of acetaldehyde with ethanol generates acetal (1,1-diethoxyethane) [36]. Other aroma compounds, such as acids and furans, although present in trace amounts, also significantly influence the aroma profile of Laobaigan Baijiu.

**Figure 6:**
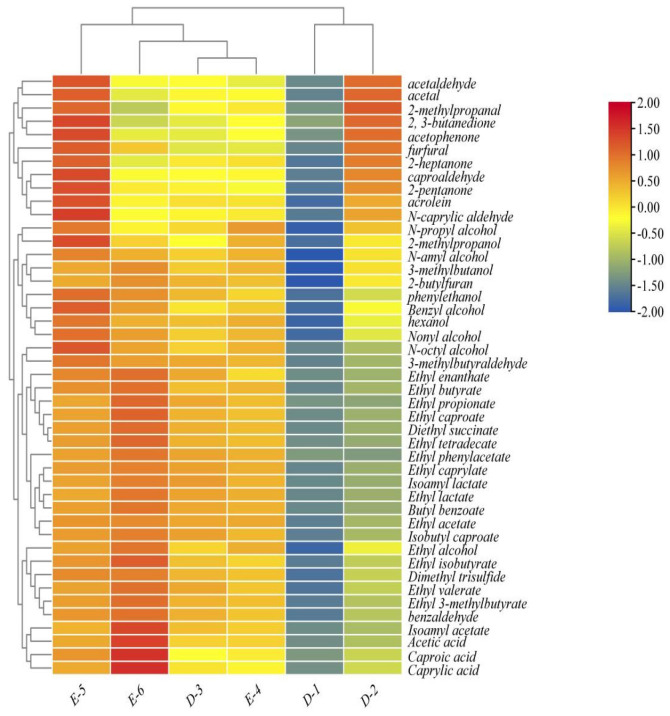
Heatmap and cluster analysis of volatile flavor compounds at different fermentation stages of Laobaigan Baijiu.

The volatile profiles and their dynamic changes observed in Laobaigan Baijiu fermentation are consistent with patterns reported in other solid-state fermentation systems, suggesting that the role of batch progression in shaping flavor may be a common phenomenon [6,17,37,38]. To facilitate a more intuitive comparison of different flavor compounds, Figure 6 presents a heatmap of the detected flavor compounds, followed by cluster analysis. A hierarchical clustering heatmap indicated that Laobaigan Baijiu samples from Dacha and Ercha fermentation differ markedly in their flavor compound composition. The sample clustering results indicated that fermentation cycle is an important factor influencing flavor composition. Samples from various fermentation phases of Laobaigan Baijiu formed two main clusters. One cluster comprised **D-1** together with **D-2** from the Dacha fermentative period, which exhibited relatively low and similar contents of most flavor compounds. The second cluster included the Dacha sample **D-3** and all Ercha samples (**E-4**, **E-5**, **E-6**). Among these, **D-3** had contents of key compounds (e.g., esters, higher alcohols, and organic acids) that were very similar to those of the Ercha samples, suggesting that the flavor profile of this stage was transitioning toward that of Ercha Baijiu. In the Ercha fermentation stage, **E-5** and **E-6** exhibited high similarity, and the fermentation consistency was higher than that of the Dacha stage. Furthermore, based on compound clustering, key flavor compounds in Ercha Baijiu—including esters (ethyl acetate, ethyl hexanoate, ethyl butyrate, etc.), higher alcohols (3-methylbutanol), and organic acids (hexanoic acid, acetic acid)—are concentrated in the red high-expression regions of the heatmap, and their contents are higher than those in **D-1** and **D-2**. These results indicate systematic differences in flavor composition between Dacha and Ercha Baijiu and reveal that **D-3**, although a Dacha sample, has a flavor profile closer to that of Ercha. This provides a visual basis for further analysis of fermentation mechanisms across different fermentation rounds and batch-to-batch variations. Collectively, these flavor compounds lend Laobaigan Baijiu its characteristic flavor.

### 3.4. Correlation Analysis Between Bacteria and Volatile Flavor Compounds in Hengshui Laobaigan Baijiu

**Figure 7:**
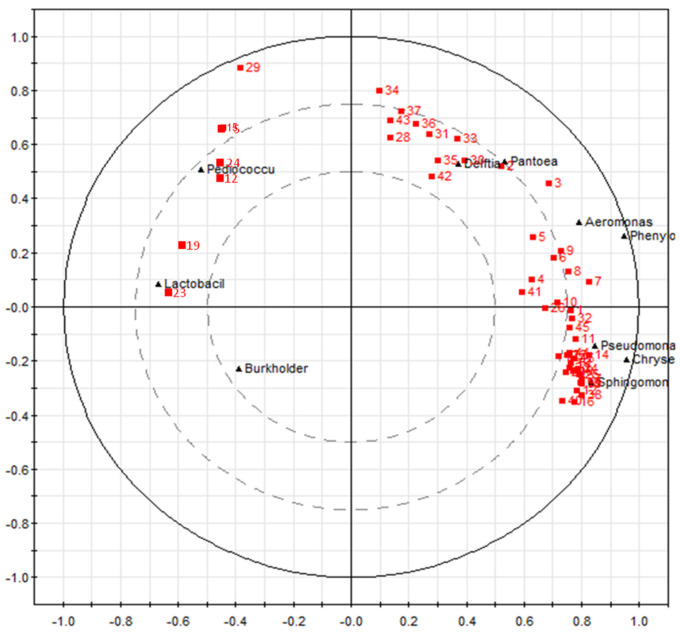
Correlation of bacterial microbiota with 45 flavor compounds.

To further elucidate how dominant bacterial communities affect the formation of volatile flavor compounds during Hengshui Laobaigan Baijiu fermentation, this study constructed a partial least squares regression (PLS) model, with 10 dominant bacterial genera as independent variables (X) and 45 volatile compounds as dependent variables (Y), to explore the correlation between bacterial community structure and flavor compounds. The model was built using 30 samples (six fermentation stages × five biological replicates). Leave-one-out cross-validation yielded a cumulative R^2^Y of 0.81 and a cumulative Q^2^ of 0.62, indicating good explanatory and predictive power. A permutation test (200 permutations) confirmed the model validity (*p* < 0.05), with no overfitting. Variable importance in projection (VIP) scores identified key genera contributing to flavor formation: *Pseudomonas* (VIP = 1.72), *Sphingomonas* (VIP = 1.68), *Lactobacillus* (VIP = 1.51), *Delftia* (VIP = 1.12), and *Pantoea* (VIP = 1.05) had VIP values > 1, indicating their significant contributions to the flavor profile. As shown in Figure 7, most variables fall within the 50–100% confidence interval. The shorter the spatial distance between variables in the projection space, the stronger the positive correlation; conversely, a greater distance or opposite direction indicates a weaker or negative correlation [39].

*Lactobacillus* is mainly located on the left side of the plot and shows a positive correlation with ethyl lactate (**19**) and isoamyl lactate (**23**). Ethyl lactate is a core component of the typical flavor of Hengshui Laobaigan Baijiu, while isoamyl lactate also contributes a soft fruity and ester-like aroma [40]. *Pseudomonas* is predominantly distributed in the right region of the plot and exhibits a significant positive correlation with fruity and floral esters, including ethyl acetate (**11**), ethyl isobutyrate (**13**), ethyl butyrate (**14**), ethyl octanoate (**21**), isobutyl hexanoate (**22**), and ethyl phenylacetate (**26**). *Pseudomonas* has been associated with amino acid metabolism and lipid degradation. It may participate in the formation of medium- and short-chain esters during flavor development in Hengshui Laobaigan Baijiu and could be an important microbial group contributing to fruity aroma, ester richness, and flavor complexity [41]. *Sphingomonas* is predominantly distributed in the right region and is spatially closely linked to a variety of fruity and floral esters, including isoamyl acetate (**16**), ethyl pentanoate (**17**), isobutyl hexanoate (**22**), ethyl phenylacetate (**26**), and ethyl myristate (**27**), as well as to organic acids such as acetic acid (**38**), hexanoic acid (**39**), octanoic acid (**40**), and to benzaldehyde (**44**), exhibiting a significant positive correlation. Meanwhile, this genus also tends to cluster with other surrounding flavor compounds, including alcohols and esters. Thus, *Sphingomonas* may be widely involved in the metabolic synthesis of medium- and long-chain fatty acid esters, organic acids, and aromatic compounds, thereby potentially contributing to fruity notes, ester richness, and acid balance in Baijiu. Small-molecule aldehydes and ketones, including acrolein (**30**), octanal (**33**), 2-pentanone (**35**), and furfural (**42**), are predominantly distributed in the upper right region of the plot and exhibit an extremely strong correlation with *Delftia*. Furthermore, the neighboring genus *Pantoea* also shows extremely strong correlations with 1-propanol (**2**), acrolein (**30**), and octanal (**33**). These two genera were abundant in the early stage of community succession and may be involved in the degradation of raw materials and the production of flavor precursors, thereby potentially providing a material basis for the formation of characteristic flavor compounds in later stages [42].

These results indicate that the flavor complexity of Hengshui Laobaigan Baijiu is not determined by a single microbial community. Instead, it is achieved through the sequential action of different microbial communities across different fermentation stages. *Delftia* and *Pantoea* degrade macromolecular substances in raw materials during the early fermentation stage, producing amino acids, short-chain fatty acids, and reducing sugars. These intermediates are then sequentially utilized by later-stage microbial communities, serving as precursors for the synthesis of characteristic esters. *Lactobacillus* not only directly synthesizes lactate esters but also lowers the pH of the fermented grains by producing large quantities of acid. This environmental change inhibits acid-sensitive microbial communities while simultaneously creating more favorable conditions for esterification [43]. The metabolic activity of *Lactobacillus* alters the surrounding microenvironment, which in turn reinforces its dominance. In the Ercha fermentation stage (**E-4** to **E-6**), the abundance of *Lactobacillus* decreased compared to that in the late Dacha stage (**D-3**). At this stage, *Pseudomonas* and *Sphingomonas* began their metabolic activities, and although they were not abundant, they were capable of synthesizing various fruity and floral esters in their adapted environment. Dacha may primarily contribute to establishing the fundamental flavor framework, whereas Ercha may mainly contribute to enhancing flavor complexity and layering. Thus, the rich flavor of Hengshui Laobaigan Baijiu results from the synergistic and complementary interactions among different microbial communities. The fermentation environment (acidity, ethanol, oxygen) may serve as an important factor influencing microbial community succession and functional transitions. Therefore, strategies for regulating the quality of Laobaigan Baijiu can be based on the combined action of multiple microbial communities. Future process optimization can focus on precisely controlling fermentation environmental parameters at different stages. For example, the rate of acidity increase could be suitably controlled during the late Dacha stage (**D-3**), or temperature and aeration conditions could be adjusted during the Ercha stage to enhance the activity of microbial communities responsible for synthesizing fruity esters, thereby specifically improving the flavor complexity of Laobaigan Baijiu. The approach presented here—combining HTS, HS-SPME-GC-MS, and PLSR—can be readily adapted to study other solid-state fermented products where batch-to-batch variation significantly influences quality [6,17,37,38].

## 4. Conclusions

This study systematically elucidated the bacterial community structure and its intrinsic link to the formation of volatile flavor compounds across different fermentation stages of Hengshui Laobaigan Baijiu. The results suggest that fermentation rounds and stages may be important factors influencing bacterial community succession and flavor formation. During Dacha fermentation, the relative abundance of *Lactobacillus* reached 92.48% at the **D-3** stage. During the Ercha fermentative stage, relative abundances of *Pseudomonas*, *Chryseobacterium*, and *Delftia* rose markedly, and the Ercha stage also exhibited higher levels of key flavor compounds (e.g., esters, higher alcohols, organic acids) than the Dacha stage did. The concentrations of ethyl acetate, ethyl lactate, and ethyl hexanoate peaked at the end of fermentation (**E-6**), reaching their highest levels among all stages.

Correlation analysis further revealed the co-variation patterns between bacterial communities and flavor compounds. *Lactobacillus* was positively correlated with ethyl lactate and isoamyl lactate, suggesting that it may be involved in the production of organic acids and might thereby contribute to esterification processes. *Pseudomonas* shows significant positive correlations with ethyl acetate, ethyl isobutyrate, ethyl butyrate, ethyl octanoate, isobutyl hexanoate, and ethyl phenylacetate. *Sphingomonas* was significantly positively correlated with isoamyl acetate, ethyl pentanoate, isobutyl hexanoate, ethyl phenylacetate, and ethyl myristate, as well as with organic acids (acetic acid, hexanoic acid, octanoic acid) and benzaldehyde. *Delftia* exhibits an extremely strong correlation with acrolein, octanal, 2-pentanone, and furfural. The genus *Pantoea* also shows extremely strong correlations with 1-propanol (**2**), acrolein (**30**), and octanal (**33**). For the **D-3** specimen within the Dacha fermentation stage, its flavor profile closely resembles that of Ercha, and its bacterial community composition also approaches that of Ercha, indicating that bacterial community succession is continuous during the late fermentation stage. In addition, considering that fermentation time, batch transition, and raw material renewal changed simultaneously in this design, the results revealed a descriptive pattern of microbial community succession at different fermentation stages: in the middle and late Dacha stages, *Lactobacillus* was the dominant genus, which may contribute to the foundation of lactate esters; in the Ercha stage, the relative abundance of *Lactobacillus* decreased, while *Pseudomonas* and *Sphingomonas* became more abundant, suggesting their possible involvement in the production of fruity and floral esters. The flavor of Laobaigan Baijiu is complex, and it is plausible that the metabolic functions of the microbial community display a degree of specialization and synergy. However, it should be noted that these interpretations are based on genus-level taxonomic resolution. Functional capabilities can vary substantially among species or strains within the same genus. Therefore, the proposed functional roles should be considered as hypotheses that require further validation using higher-resolution methods (e.g., metagenomics or strain isolation).

This study established a correlation network between bacterial communities and flavor compounds across different fermentation stages of Laobaigan Baijiu, extending previous findings on microbiota–flavor associations in other Baijiu types. It suggested that core functional microbiota may play important roles in key flavor formation, provided theoretical support for elucidating standardized production and quality improvement of Laobaigan Baijiu, and offered targeted microbiota (e.g., enhancing *Pseudomonas* activity in Ercha) and metabolic nodes (e.g., the ratio of ethyl lactate to fruity esters). These findings provide potential targets for process regulation. Future research may integrate metagenomics or metabolomics to further validate the key metabolic pathways involved in ester and higher alcohol formation. Such insights could then guide the development of optimization strategies to enhance the accumulation of these compounds in Ercha Baijiu, thereby improving flavor quality. In summary, although this case study focused on Hengshui Laobaigan Baijiu, the integrated analytical workflow (16S rRNA sequencing + HS-SPME-GC-MS + PLSR) and the identified bacterial-flavor associations (e.g., *Lactobacillus* with lactate esters, *Pseudomonas* with fruity esters) can serve as a reference for studying flavor formation in other traditional solid-state fermentation systems. This work thus contributes not only to the optimization of Laobaigan Baijiu production but also to the broader field of food fermentation microbiology and flavor regulation.

## Figures and Tables

**Table 1:** Bacterial alpha diversity indices of fermented grain samples.

Sample ID	Observed Species	Chao1	Shannon	Simpson	Goods_Coverage
D-1	173.8 ± 3.96 ^d^	225.1 ± 18.1 ^de^	3.401 ± 0.376 ^d^	0.744 ± 0.048 ^d^	0.99853 ± 0.00018
D-2	84.4 ± 12.1 ^b^	114.4 ± 14.1 ^b^	1.755 ± 0.380 ^b^	0.464 ± 0.114 ^b^	0.99914 ± 0.00013
D-3	60.4 ± 4.0 ^a^	78.7 ± 6.2 ^a^	0.629 ± 0.053 ^a^	0.151 ± 0.020 ^a^	0.99942 ± 0.00008
E-4	159.6 ± 21.1 ^d^	189.8 ± 18.4 ^cd^	3.522 ± 0.351 ^d^	0.784 ± 0.044 ^d^	0.99884 ± 0.00018
E-5	188.6 ± 11.6 ^e^	228.8 ± 24.8 ^e^	3.382 ± 0.504 ^d^	0.742 ± 0.082 ^d^	0.99850 ± 0.00022
E-6	121.4 ± 12.9 ^c^	168.5 ± 31.9 ^c^	2.451 ± 0.321 ^c^	0.650 ± 0.072 ^c^	0.99889 ± 0.00020

Notes: 1: Data are mean ± SD (n = 5); 2: Different superscript letters in the same column indicate significant differences among fermentation stages based on pairwise comparisons using Tukey’s HSD post hoc test (*p* < 0.05); 3: Overall ANOVA *p*-values for all α-diversity indices were <0.001; 4: Goods coverage values were all >0.998, indicating sufficient sequencing depth; therefore, no superscript letters are shown for this index.

**Table 2:** Volatile flavor compounds at various fermentation phases of Laobaigan Baijiu.

No.	Compounds	Concentration (μg/L)					
		D-1	D-2	D-3	E-4	E-5	E-6
	Alcohols						
1	Ethanol	3.341 ± 0.302	29.893 ± 2.512	38.780 ± 2.673	44.657 ± 4.276	47.785 ± 4.365	53.451 ± 4.174
2	n-Propanol	ND	1.023 ± 0.031	0.934 ± 0.063	1.178 ± 0.102	1.309 ± 0.104	0.824 ± 0.083
3	2-Methylpropanol	ND	0.102 ± 0.072	0.092 ± 0.026	0.131 ± 0.082	0.182 ± 0.074	0.114 ± 0.071
4	3-Methylbutanol	2.012 ± 0.014	20.438 ± 2.421	22.091 ± 2.437	23.598 ± 2.379	24.781 ± 2.421	26.900 ± 2.724
5	n-Pentanol	ND	0.093 ± 0.025	0.101 ± 0.063	0.112 ± 0.074	0.131 ± 0.054	0.113 ± 0.068
6	1-Hexanol	0.207 ± 0.047	0.903 ± 0.082	1.238 ± 0.122	1.301 ± 0.141	1.526 ± 0.153	1.290 ± 0.124
7	1-Octanol	ND	0.112 ± 0.076	0.320 ± 0.058	0.368 ± 0.047	0.520 ± 0.062	0.392 ± 0.062
8	1-Nonanol	ND	0.089 ± 0.012	0.134 ± 0.057	0.147 ± 0.084	0.187 ± 0.072	0.161 ± 0.053
9	Benzyl alcohol	ND	0.045 ± 0.007	0.053 ± 0.007	0.060 ± 0.008	0.087 ± 0.012	0.071 ± 0.008
10	Phenylethyl alcohol	ND	0.023 ± 0.002	0.043 ± 0.004	0.038 ± 0.003	0.056 ± 0.007	0.050 ± 0.006
	Esters						
11	Ethyl acetate	0.023 ± 0.002	3.901 ± 0.312	13.784 ± 1.783	13.672 ± 1.745	14.778 ± 1.772	15.321 ± 1.527
12	Ethyl propionate	ND	0.023 ± 0.002	0.243 ± 0.075	0.221 ± 0.072	0.245 ± 0.057	0.314 ± 0.085
13	Ethyl isobutyrate	ND	0.093 ± 0.005	0.210 ± 0.085	0.191 ± 0.073	0.249 ± 0.072	0.302 ± 0.083
14	Ethyl butyrate	ND	0.539 ± 0.062	1.934 ± 0.172	2.013 ± 0.224	2.352 ± 0.252	2.654 ± 0.243
15	Ethyl 3-methylbutanoate	ND	0.099 ± 0.024	0.265 ± 0.063	0.253 ± 0.058	0.289 ± 0.058	0.343 ± 0.074
16	Isoamyl acetate	0.011 ± 0.001	0.897 ± 0.083	3.125 ± 0.426	2.908 ± 0.285	3.280 ± 0.282	4.905 ± 0.427
17	Ethyl pentanoate	ND	0.298 ± 0.048	0.689 ± 0.062	0.607 ± 0.072	0.698 ± 0.075	0.832 ± 0.081
18	Ethyl hexanoate	0.020 ± 0.002	1.381 ± 0.175	6.459 ± 0.584	6.120 ± 0.627	6.990 ± 0.672	8.908 ± 0.903
19	Ethyl lactate	ND	0.674 ± 0.053	3.278 ± 0.461	3.099 ± 0.467	3.201 ± 0.427	3.864 ± 0.431
20	Ethyl heptanoate	ND	0.050 ± 0.006	0.243 ± 0.054	0.189 ± 0.028	0.278 ± 0.038	0.305 ± 0.068
21	Ethyl octanoate	0.011 ± 0.001	0.572 ± 0.072	2.680 ± 0.302	2.534 ± 0.245	2.723 ± 0.257	3.024 ± 0.428
22	Isobutyl hexanoate	ND	0.034 ± 0.002	0.119 ± 0.064	0.107 ± 0.038	0.121 ± 0.125	0.134 ± 0.081
23	Isoamyl lactate	ND	0.063 ± 0.007	0.339 ± 0.068	0.307 ± 0.082	0.329 ± 0.073	0.378 ± 0.072
24	Ethyl benzoate	ND	0.058 ± 0.006	0.302 ± 0.082	0.289 ± 0.036	0.313 ± 0.062	0.367 ± 0.075
25	Diethyl succinate	ND	0.108 ± 0.036	0.502 ± 0.071	0.478 ± 0.085	0.539 ± 0.071	0.656 ± 0.082
26	Ethyl phenylacetate	ND	ND	0.052 ± 0.006	0.049 ± 0.006	0.053 ± 0.007	0.063 ± 0.007
27	Ethyl myristate	ND	0.010 ± 0.003	0.061 ± 0.006	0.058 ± 0.006	0.067 ± 0.007	0.082 ± 0.007
	Aldehydes						
28	Acetaldehyde	ND	0.132 ± 0.072	0.067 ± 0.007	0.056 ± 0.006	0.142 ± 0.076	0.063 ± 0.006
29	2-Methylpropanal	ND	0.209 ± 0.064	0.098 ± 0.008	0.109 ± 0.036	0.199 ± 0.058	0.047 ± 0.005
30	Acrolein	ND	0.209 ± 0.062	0.168 ± 0.065	0.165 ± 0.064	0.278 ± 0.041	0.152 ± 0.084
31	Hexanal	ND	0.301 ± 0.026	0.175 ± 0.086	0.179 ± 0.086	0.367 ± 0.034	0.167 ± 0.082
32	3-Methylbutanal	ND	0.013 ± 0.003	0.050 ± 0.006	0.047 ± 0.005	0.060 ± 0.007	0.052 ± 0.007
33	Octanal	ND	0.090 ± 0.008	0.061 ± 0.006	0.065 ± 0.006	0.125 ± 0.073	0.057 ± 0.007
	Ketones						
34	2,3-Butanedione	0.012 ± 0.003	0.132 ± 0.074	0.052 ± 0.006	0.064 ± 0.007	0.148 ± 0.082	0.040 ± 0.005
35	2-Pentanone	0.034 ± 0.006	0.209 ± 0.062	0.148 ± 0.063	0.136 ± 0.057	0.249 ± 0.048	0.152 ± 0.068
36	2-Heptanone	0.022 ± 0.003	0.200 ± 0.032	0.136 ± 0.049	0.141 ± 0.073	0.217 ± 0.054	0.108 ± 0.036
37	Acetophenone	0.016 ± 0.005	0.238 ± 0.068	0.103 ± 0.043	0.121 ± 0.069	0.266 ± 0.074	0.104 ± 0.041
	Acids						
38	Acetic acid	0.007 ± 0.001	0.010 ± 0.003	0.016 ± 0.003	0.016 ± 0.002	0.018 ± 0.005	0.023 ± 0.006
39	Hexanoic acid	0.012 ± 0.003	0.023 ± 0.003	0.031 ± 0.005	0.034 ± 0.005	0.047 ± 0.006	0.062 ± 0.007
40	Octanoic acid	0.014 ± 0.002	0.025 ± 0.003	0.034 ± 0.005	0.032 ± 0.005	0.041 ± 0.006	0.056 ± 0.006
	Others	ND	0.091 ± 0.008	0.112 ± 0.043	0.107 ± 0.036	0.116 ± 0.065	0.126 ± 0.072
41	2-Butylfuran	0.012 ± 0.004	0.278 ± 0.058	0.123 ± 0.072	0.126 ± 0.068	0.301 ± 0.026	0.203 ± 0.037
42	Furfural	ND	1.001 ± 0.142	0.531 ± 0.074	0.521 ± 0.076	1.031 ± 0.092	0.420 ± 0.065
43	Acetal	ND	0.034 ± 0.007	0.070 ± 0.007	0.065 ± 0.007	0.076 ± 0.008	0.085 ± 0.010
44	Benzaldehyde	0.011 ± 0.003	0.127 ± 0.074	0.264 ± 0.062	0.253 ± 0.046	0.311 ± 0.076	0.324 ± 0.074
45	Dimethyl trisulfide	ND	0.091 ± 0.008	0.112 ± 0.037	0.107 ± 0.026	0.116 ± 0.062	0.126 ± 0.075

Note: Data are presented as mean ± SD (n = 5). “ND” indicates not detected (below the limit of detection). Concentrations are semi-quantitative estimates based on internal standard calibration.

## Data Availability

The original contributions presented in this study are included in the article. Further inquiries can be directed to the corresponding author.
